# The role of the social environment in inclusive sports participation—Identifying similarities and challenges in athletes with and without Intellectual Disabilities through coaches’ eyes: A qualitative inquiry

**DOI:** 10.1371/journal.pone.0280379

**Published:** 2023-01-11

**Authors:** Kandianos Emmanouil Sakalidis, Anastasia Fadeeva, Florentina Johanna Hettinga, Fiona Chun Man Ling

**Affiliations:** 1 Department of Sport Exercise and Rehabilitation, Faculty of Health and Life Sciences, Northumbria University, Newcastle upon Tyne, United Kingdom; 2 Violence and Society Centre, City, University of London, London, United Kingdom; Mugla Sitki Kocman University: Mugla Sitki Kocman Universitesi, TURKEY

## Abstract

Despite the efforts of mainstreaming in sports, inclusive participation of people with Intellectual Disabilities (ID) in sports remains challenging. In sports settings, the social environment may influence the motivation of athletes and promote (or restrict) inclusive sports participation of athletes with ID. Thus, this study aims to explore the motivations of athletes and coaches and to investigate the role of the social environment in sports participation of athletes with and without ID. Coaches who coach athletes with ID (n = 11), athletes without ID (n = 13) and both groups (n = 2) were involved in semi-structured interviews. From the inductive thematic analysis three themes were identified for the sports motivations of athletes with ID (Sport-related Progression, Social Interaction, Positive Emotions), two for their coaches’ motivations (Help others, Personal and Professional Development) and four for coaching practices toward athletes with ID (Psychological and Life skills development, Building Meaningful Relationships, Behaviour adaptations and Participation-focused). Moreover, ‘Teammates and Opponents’ and ‘Family’ were classified as social agents that influence the sport participation of athletes with ID. Coaches of athletes without ID reported additional themes about their athletes’ motivations (Health-related Reasons), their coaching motivations (Career aspirations) and their coaching practices towards athletes without ID (Performance-focused). The motivations of athletes and the practices of coaches could promote sports participation of athletes with ID, but more work is needed to address athletes’ sports motivations and overcome the able-ist attitudes and the social oppression that may make their inclusion in sports more challenging. These sports participation barriers and facilitators could provide direction to stakeholders for developing inclusive sports pathways to people with ID.

## Introduction

In recent years, there is growing emphasis on the integration of non-disability and disability sports organisations, known as mainstreaming, due to the continued global call for equal opportunities in sports [1]. To champion the mainstreaming movement, it is crucial to promote inclusivity within the sports environment. Inclusive practices refer to the provision of enhanced opportunities to people with disabilities to participate in the exercise and sport activity of their choice [2]. A better understanding of the needs, motivation, and social environment of athletes, including the active involvement of coaches and parents, are pivotal in driving inclusivity in sports settings [3, 4]. While there is considerable understanding of the role of social agents for athletes without disabilities [5], such is relatively understudied in athletes with Intellectual Disabilities (ID) [6]. The lack of appreciation of the similarities and differences in athletes with and without ID, and their interactions with their social environment, may have hindered progress of inclusivity within the sport settings, as well as sport engagement for individuals with ID [7].

People with ID are dealing with limitations in intellectual capacities (IQ ≤ 70) and adaptive skills (conceptual, practical, social skills). These limitations can negatively influence sports-related skills like decision-making, self-regulation (e.g., goal setting, self-reactions) and learning by experience, resulting in deteriorated sport performance and development [8, 9]. For instance, people with ID demonstrate reduced technical proficiency [9] and inadequate pacing behaviour in competitive races [10]. People with ID also have difficulties in understanding and executing the instructions of coaches [10]. Additionally, they may misinterpret the behaviour of other people, and/or inadequately convey messages across, which can hinder social interaction [11]. Consequently, people with ID might respond differently to environmental cues [12, 13] impacting on their interaction with their coaches, teammates (e.g., during training sessions) and/or their opponents. In head-to-head competitions for instance, the ability to appropriately respond to the actions of their opponents, a relevant skill for optimal performance [14], is underdeveloped in athletes with ID [10]. Apart from their challenging behaviours, people with ID are also dealing with anxiety, decreased confidence, and social phobia, all of which can influence sports motivation [15, 16]. Because of these cognitive deficits and psychological barriers, people with ID may become dependent on the support of others in their daily lives (e.g., coaches, parents, carers) [17]. For example, as people with ID are less proficient in self-regulation, the social environment can step in and help them set goals, give feedback, and alter their affective reactions [5, 10]. Thus, the social environment is critical in influencing sports motivation and promoting and/or restricting sports participation of this population [18, 19].

For this reason, we are interested in understanding the motivation and the social environment for athletes with ID, and compare it with athletes without ID, in order to understand the challenges in adopting inclusive practices in the mainstreaming initiatives. Although there is a growing interest in the intricate role of the social agents in the inclusive sport environment [4, 20], research is still scarce. In this study, we focus on the lived experiences of coaches because coaches play a key role in enhancing athletes’ sport-related skills (e.g., self-regulation), creating an exercise motivational climate and promoting the sports engagement of the athletes [21, 22]. We will consider how the athletes’ and coaches’ sports-related motivations shape their relationships and guide the sport behaviour of the athletes’ [23], a gap in literature, especially in the ID population, that warrants investigation. Hence, a comparison in motivations between coaches, and the motivations of their athletes (with and without ID), as well as the behaviour of coaches will provide insights into the needs and challenges of creating a sustainable inclusive sport environment [1].

In view of the above, the aims of this study are to i) investigate athletes’ motivations for sports participation (as perceived by coaches), ID) explore coaches’ coaching motivations, ID) gain a better understanding of the coaching practices and iv) explore the role of the social environment in athletes’ sports participation and development based on coaches’ reports. Perspectives of coaches from ID and non-ID athletes will be compared.

## Materials and methods

### Participants and recruitment

After gaining ethical approval by the Institutional Ethics Board, participants were recruited from January until May of 2021 through a combination of criterion-based and maximum variation sampling strategy [24] in order to capture key variations in participants’ coaching experience, age, and type of sport. With regards to the inclusion criteria, we recruited coaches of athletes with and without ID who were fluent in English, had at least one year of coaching experience, and their athletes were adults or adolescents (above 12 years old) who met the criteria for diagnosis of ID: limitations in intellectual and adaptive functioning with an IQ ≤ 70, limitations in social, conceptual, and practical skills, and manifested before the age of 18 years old [8]. For comparability purpose, all athletes were in the ‘participation’ or ‘performance’ stage of sport development where focus is on sports skill acquisition, and with some experience in competition at local and/or regional level [25, 26]. Participants were recruited through websites, social media, and emails via charities, sports organisations, and sports clubs. A code was given to each participant; therefore, the collected and stored data did not reflect any personal information of the participants. Moreover, researchers did not store email addresses and/or phone numbers which were essential for the interviews. Twenty-six coaches agreed to participate by signing a written consent form (aged 21–79 years, coaching experience between one and 43 years, types of sport include basketball, archery, badminton, athletics, boccia, cycling, swimming, tennis, weightlifting, rugby, football, gymnastics, goalball, cricket, netball, and table tennis; see Table 1 for detailed demographics information). Data collection was completed when the researchers considered no additional information could be yielded from additional interviews.

**Table 1:** Participants’ demographic information.

Coaching group	N	Male (%)	Age (< 40yrs %)	Years of coaching experience (<20yrs %)
Athletes with ID	11	72.72	54.54	72.72
Athletes without ID	13	61.53	38.46	61.53
Athletes with and without ID	2	50	50	50

N = Number of coaches.

### Procedure

All participants were involved in semi-structured interviews conducted by the first author (KS) via video-calling platforms (Skype or Zoom; n = 24) or telephone (n = 2). Each interview lasted about an hour and participants were asked about their coaching motivations (e.g., “What motivates you to continue coaching your sport?”), their relationship with their athletes (e.g., “How would you describe your relationship with your athletes?”) and interpersonal styles (e.g., “How would you characterise yourself as a coach?”). Coaches also gave opinions about their athletes’ motivations (e.g., “Why are your athletes participating in sports?”) and athletes’ social environment in sports (e.g., “How are your athletes behave to one another during training and competition?”). The interviewer used the interview guide flexibly, and follow-up questions were used during the process to elicit richer data [24]. All interviews were recorded and transcribed verbatim with the assistance of an online transcription software (Otter.ai) for data analysis.

### Analytic procedure

Transcripts were analysed using a reflexive thematic analysis with an inductive orientation, an iterative and progressive method that allows the authors to identify and provide a detailed analysis of patterns across the data set [27, 28]. Initially, the first author (KS) immersed in the data, making notes of preliminary patterns, and generating initial ideas. Codes were then generated from the transcripts using the NVivo software and finally, grouped into themes [27, 29]. KS then discussed the structure and the content of the themes with the second author (AF), who also coded the transcripts. AF acted as a ‘critical friend’, questioning KS’s assumptions, codes, and themes to promote reflection and ensure further development of meaningful themes [24]. To improve fidelity, KS tried to collect data from coaches with diverse characteristics (in terms of coaching experience, age, and type of sport) and to become familiar with the culture of participants [30, 31]. The analysis was underpinned by the constructivist paradigm, individuals construct knowledge through their own experience and interaction with the world [32]. In terms of the coaching experiences and perceptions of participants, we interpret them as the reality of the experiences of coaches while at the same time we take into consideration the cultural, political, and historical context in which they occur [32]. The investigators were aware that their own experiences and perspectives could influence the research process. For instance, the first author (KS) had an ’insider’ perspective of the culture of coaches (he was a coach of non-British athletes with and without ID, but prior the interviews, he also tried to become more familiar with the culture of the coaches in the United Kingdom). His priory knowledge helped him ask more insightful and meaningful questions, but he might be overly sympathetic to the coaches’ culture [31]. To effectively deal with this issue, KS used open-ended questions and nonleading language during the interviews [30]. During the data analysis, the discussions with the second (AF) and the last author (FL) who had ’outsider’ perspectives (less familiar with the culture of coaches) ensured that the results were interpreted based on the perspective of participants [31]. Moreover, KS recorded reflective notes (memoing) to manage his own perspective and ensure fidelity [30]. With the above practices, the investigators attempted to articulate their positionality and avoid systematic and conscious bias, so that the results and their interpretations reflect the lived experience of the participants as much as possible [31].

## Results

### Athletes’ sports motivations (as perceived by coaches)

With regards to sports motivations of athletes with and without ID, three themes were identified and categorised into ‘Sport-related progression’, ‘Social Interaction’ and ‘Positive Emotions’. We identified an additional theme for the sports motivations of athletes without ID (‘Health-related reasons’).

#### Sport-related progression

According to coaches, athletes with and without ID participate in sports because they want to ‘try something different’ and because they want to learn new skills: ‘They want to learn every intricacy of basketball’ (P01, ID coach), ‘They want to see how far they can go, how good they can get’ (P22, non-ID). They also want to progress in their sport and improve their skills: ‘Athletes want to improve, to get better.’ (P19, ID coach). This sports performance progression gives them the chance to achieve something meaningful to them: ‘They can wear their cricket shirts and have this sense of pride. It is a sense of achievement for them.’ (P18, ID coach). The awareness of their progression and their sense of achievement ‘give them the confidence’ to continue training.

#### Social interaction

Participation in sports gives athletes the opportunity to interact and socialise with each other: ‘Some of them just want to come and have a chat. It’s a bit of a social group’ (P01, ID coach). For athletes with ID, sports offer an inclusive environment where ‘they can feel safe with other people that are like them, where they don’t have to feel like they’re being judged’ (P17, ID and non-ID coach). Many participants mentioned that sports are more than a social event, as they help athletes to make new friends, develop social/sport skills and a sense of belonging:

They mostly like coming to see their friends. So, I think playing the sport and gain enough confidence and gain in that friendship network game in order to gain in so many of those and soft skills that they take through sport and interacting with others (P05, ID coach).

I think the fact that you come into the gym to see your friends, this is a really big factor. Even if people aren’t amazing weightlifters, I think a main factor that keeps bringing them back is because they are training with the group (P12, non-ID coach)

#### Positive emotions

Athletes participate in sport because they ‘love it’ and it is an indispensable part of their life:

We have a football team. So, they actually take it seriously, like it’s their life… they actually want to be there, they choose to be there. And they’ve trained for it. And their mindset is that they’re playing a professional football game (P06, ID coach).

Through sports, athletes with ID experience enjoyment: ‘The thing that keeps the athlete coming back is that they are having fun’ (P1, ID coach), ‘If it wasn’t fun, they wouldn’t come’ (P10, non-ID coach). Lastly, it is important for the athletes that they can be independent and express themselves through sports:

He said, that’s the only time that he hasn’t got carers. He can just be himself in his own quietness. If he is annoyed, he can shout. If he is happy, he can be happy. And it is probably the only opportunity he gets in a week, that he can kind of just be himself and be like any other person (P04, ID coach).

#### Athletes’ sports motivations differences: Health-related reasons (athletes without ID)

According to the reports of the coaches, athletes without ID participate in sports also for health-related reasons. According to PO7 (non-ID coach) they want to ‘get fit’ and to ‘lose some weight’. Sports are a pathway for athletes to regulate their mental health as well. They relieve stress through sports and help them forget their personal problems:

Athletes found that doing archery gave them that break from whatever it caused them problems, gave them the chance to not think, because you can’t really do archery and think about anything else. (P02, non-ID coach).

#### Coaches’ coaching motivations

Having understood the motivations of the athletes in sport participation, we would like to gain an in-depth view about the coaching motivations of coaches and the extent to which they complement their athletes’ motivations. We identified two themes for coaches of athletes with and without ID—‘Help others’ and ‘Personal and Professional development’. We identified an additional theme for coaches of athletes without ID (‘Career aspirations’).

#### Help others

Coaches want to help athletes ‘reach their potential’ and develop their life skills: ‘I’m quite keen to make sure that they are allowed to make decisions’ (P18, ID coach), ‘I want my athletes to see what is possible through their body and mind, to help them be themselves’ (P22, non-ID coach). For coaches, it is important to convince athletes that they can achieve something:

I thought the best thing to do with D. is to give him a cricket bat. We got to this point where ‘bang’, he hit the ball… That was a small win, but in his world, it was a huge win. It was emotionally very powerful. And it’s still even to this day, one of the things that continues to motivate me into doing what we’re doing, because it makes a difference (P18, ID coach).

#### Personal and professional development

Coaches reported that they ‘love the sport’ that they are coaching. They also ‘enjoy’ interacting with their athletes, as this interaction brings the best out of them personally and professionally:

I love it and you get so much out of it. As a coach, it’s really rewarding. And you have more fun. I feel like the students bring the best out of me as a coach as well as a person… that makes your job worthwhile. You do not mind putting in the extra work and the extra enthusiasm into your job. (P06, ID coach).

Coaches are also intricated by the psychological and the social benefits that sports can offer them and their athletes: ‘Sports can improve your mental health your well-being. So that’s what I really love about sports coaching’ (P19, ID coach), ‘I love the aspect that the sport itself is a support network’ (P16, non-ID coach).

#### Coaches’ coaching motivations differences: Career aspirations (non-ID coaches)

Apart from personal and professional development, coaches of athletes without ID are also driven by their career aspirations. ‘I want to have respect in my local area (P23, non-ID coach). For example, one female participant wants to become one of the first female coaches in a male-dominant sport:

Olympic weightlifting is a very male dominant sport, just in general, not just in the coaching. One of my motivations of becoming a coach was because I didn’t know any female coaches when I was learning, I was always taught by males. And I just thought I would love to try and really drive the participation of females in this male dominance sport (P12, non-ID coach).

#### Coaching practices towards athletes with and without ID

Having a clearer picture of the motivations of athletes in sport participation and coaches’ coaching motivations, we would like to the understand how coaches’ motivations may influence coaching practices, and in turn, shape coach-athlete relationship. With regards to coaching practices towards athletes with and without ID (second aim), three themes were identified—‘Psychological and life skills development’, ‘Building Meaningful relationships’ and ‘Behaviour adaptation’. We identified an additional theme for the coaching practices towards athletes with ID (‘Participation-focused’) and without ID (‘Performance-focused’).

#### Psychological and life skills development

For coaches, sports are an excellent opportunity to help their athletes acquire the necessary soft skills for optimal functioning in daily life: ‘I give them a lot of autonomy, I give them a lot of choices’ (P15, non-ID coach). Coaches encourage their athletes to be independent, engage them in the learning process and give them responsibilities and options:

I’m quite keen to make sure that the students are allowed to make decisions. Because I think that those sorts of things are transferable. I think our responsibility is to get them to understand all the different options that they have… is an individual’s decision to choose one of those options (P18, ID coach).

Coaches are also trying to build values of ‘teamwork’, encouraging the interaction between their athletes and are trying to improve their social skills: ‘Get them feel comfortable talking to people, as some of them are very shy’ (P03, ID coach), ‘I want them to engage with each other and work together as a team. Otherwise, gymnastics could be a quite lonely sport’ (P15, non-ID). Moreover, they are trying to make them aware that in sports, as in life, failure is an option and athletes have to ‘learn to win and lose’ and that they can be better persons through sports: ‘Our coaching philosophy should be around making better people’ (P18, ID coach).

### Building meaningful relationships

Coaches are constantly trying to connect with their athletes and build meaningful relationships. ‘They respect me because I respect them.’ (P15, non-ID coach) and they are trying to find ‘what works well for them’. They are also trying to interact and conversate with their athletes as much as possible, offer them a safe training environment and gain their trust:

The trust is the most important… one of the things that I’ve always say to people: ‘try yourself to be him, how would you do it yourself?’. They have to trust you. You will never ask them to do something, you wouldn’t do yourself, right? (P08, ID coach).

A number of coaches highlighted the importance to be a ‘role model’ of their athletes and act as a family member to them: ‘I’m like a father figure to then, care for them, look after them’ (P08, ID coach). Coaches are also trying to be ‘friendly’, ‘funny’ and ‘lovable’ to their athletes.

However, a meaningful relationship is also based on setting clear boundaries to facilitate optimal functioning of this relationship. Thus, coaches are trying to run the sessions as smoothly as possible, avoid conflicts and promote a safe environment. To achieve that, they require from their athletes to ‘follow rules’ and ‘have good manners’.

#### Behaviour adaptations

Coaches reported the importance to adapt their sessions: ‘I’m quite flexible as to what the training can be, depending on how they feel’ (P20, ID coach) and interact with flexibility with the athletes: ‘What you have to do is to find out to what they respond to the best’ (P08, ID coach). Being aware of athletes’ behaviour issues could help guide coaches’ behaviour and coaching approach: ‘You can trigger someone if you are too instructive and authoritarian’ (P01, ID coach). ‘I have to be aware of the capacity of people to understand what I’m saying’ (P09, non-ID coach). Throughout this process, patience is key:

… although they’ve done (the drill) several times, they may want to start right from the beginning and right from the basics every time. I think you just have to be patient and, and just be aware of that, because you will have to go through things a million times. And if it doesn’t work, then there’s no point shouting or being forceful (P05, ID coach).

Their adaptations are also based on athletes’ personal needs and abilities: ‘Everyone is an individual, isn’t it? Everyone has different abilities and skills (P05, ID coach), ‘At our club we tend to tailor the coaching according to people’s ability and the time they can spend on the sport’ (P02, non-ID coach).

#### Coaching practices differences: Participation-focused (ID coaches)

A main goal from ID coaches is to engage more people with ID in sports: ‘Some of the guys, if they do 10 minutes without leaving, that’s a massive achievement’ (P01, ID coach). Thus, they are trying to make their sessions inclusive and offer to people with ID different sports opportunities: ‘The opportunity for them to be able to participate in whatever activity it may be, whether it’s actually a proper competition, or whether it’s just a fun social event’ (P17, ID and non-ID coach). For coaches, inclusion to sports have different interpretations:

Inclusion can be interpreted and can be seen in different ways. Because if you’re working with a group of people where some people have a disability, and some haven’t, you may have an inclusive session, but it doesn’t necessarily mean that they all mixed together. I suppose to that is the door should be open for everybody. The answer to all of this is really simple. We just need to be kind to each other, just be nice to each other (P18, ID coach).

Even if inclusivity is perceived differently by the coaches, they highlighted the importance to focus on sports participation of people with ID and not on their performance: ‘This is not a performance environment, it is a participation environment’ (P18, ID coach).

#### Coaching practices differences: Performance-focused (non-ID coaches)

While both coaches of athletes with and without ID focus on the sports progression of their athletes, the latter are more performance-focused. Coaches have ‘high expectations’ from their athletes, encourage them to improve their skills and reach their sport performance potential: ‘You have to reflect on your performance, and work with the coach and create a training plan and next steps to meet your next goal.’ (P12, non-ID coach).

Coaches want their athletes to perform well in competitions and win: ‘It’s not just we’re taking part for the fun of it. We go out to win’ (P21, non-ID coach). For this reason, they ‘keep track of their sports performance progression’ and ‘prepare them physically (e.g., ‘match drills) and ‘mentally (e.g., imagery)’ for competitions.

#### Social environment and sports participation

Our third aim was to explore the role of the social environment (besides coaches) in sports participation of athletes with and without ID. Coaches perceived different social agents that may influence athletes’ sports participation and performance. We identified two groups of social agents—‘Teammates and Opponents’ and ‘Family’.

#### Teammates and opponents

Athletes with and without ID are generally friendly with each other and encourage their teammates to participate (and improve in their chosen sport) in training sessions: ‘They have a laugh and a joke to carry on’ (P20, ID coach), ‘They are committed and dedicated to helping each other’ (P22, non-ID). This positive environment enhances ‘team bonding’ and athletes’ ‘confidence’. Teammates are also trying to ‘support’ and ‘encourage’ each other during competition while at the same time they are respectful towards their opponents:

And everyone is a bit more friendly with each other, there’s a lot of mutual appreciation. Like if someone scores a basket, there’s just a lot of cheers from both sides (P01, ID coach).

However, the relationships between athletes could also be challenging. Coaches reported arguments and conflicts between teammates in training and competition: ‘I can remember a small quarrel between two of them, they were arguing over sports equipment’ (P26, ID coach), ’I had a situation where one of the girls was being bullied, and she got really upset’ (P10, non-ID coach). These behavioural problems are also present when athletes have to compete against opponents: ‘As soon as someone starts misbehaving, my athletes really start feeling a little bit anxious and stressed about it. And so that can cause bad behaviours from them as well’ (P06, ID coach). P06 (ID coach) highlighted that the athletes are ’lacking of team work experience’ and due to their social interaction difficulties, sometime ’they prefer to be on their own’ during the training session.

#### Family

Parents can facilitate the sports participation of athletes with (adults and adolescents) and without ID (only adolescents). They know the personality of athletes and can encourage them to continue participation in sports ‘The parents play a big part in athletes’ life, they know how to deal with them better than me and they encourage them to participate’ (P18, ID coach), ’One of my athletes was really proud of his park run time. And I think that’s because a member of his family was also really into park run. So, he had this kind of support’ (P07, non-ID coach). They also provide ‘positive feedback’ and ‘reinforcement’ to the athletes. For all these reasons, coaches consider their cooperation with the parents crucial:

I kind of ask parents, if anybody’s available to join. And that’s great, because that means that there is some networking going on, there is a need to be some sustainability around it. And the parental involvement is vital for that (P18, ID coach).

Nonetheless, specifically for athletes with ID, parents can be barriers for sports participation. Sports engagement of people with ID depends heavily on their parents’ support. However, it seems that some parents are not enthusiastic about their children’s involvement in sports: ‘Yeah, it’s very difficult to get parents involved. I think a lot of the time they see it as a respite.’ (P05, ID coach). According to coaches, a possible explanation for this behaviour is that the parents do not believe in their children’s abilities: ‘Parents always say, my daughter can’t do this, my daughter can’t do that (P03, ID coach) and are overprotective: ‘They don’t want to let go, they don’t want anybody else to look after them, because it’s their child’ (P04, ID coach).

## Discussion

This study investigated the sports participation motivations of athletes, the coaching motivations of coaches, and the social environment-athlete relationship and interaction in sports settings. According to the social relational model of disability (SRM), the restrictions of an activity (e.g., sports participation) can be caused by impairment (e.g., cognitive deficits) and psycho-emotional oppressions (e.g., social factors) [33]. Thus, the exploration of individual (motivations) and societal factors (social environment attitudes) of sports participation can inform coaches and stakeholders about potential strategies to create a sustainable inclusive environment for people with ID through the mainstreaming pathway. Within the sport coaching context, the relationship between coaches’ perceptions of athletes’ motivations and the enacted coaching practices and behaviours is not well-documented. As far as we know this is the first study that makes this connection in the sports environment of people with ID.

One of aims of this study was to investigate the sports participation motivations of athletes (as perceived by coaches), an individual factor that can have significant impact on athletes’ long-term participation in sports [4]. The results showed that athletes with ID, as well as athletes without ID, participate in sports mainly for intrinsic reasons (Positive emotions’, ‘Social Interaction’ and ‘Sport-related Progression’ themes), which is likely to lead to long-term engagement in sport due to greater persistence and effort [34–36]. For instance, the affective response of enjoyment that the athletes with and without ID experience (according to their coaches), can positively influence their goals and their adherence in sports [37]. Additionally, this study confirms the need of athletes with and without ID to interact with each other [38]. This attitude can promote an inclusive sport participation as teammates can boost athletes’ confidence and sense of belonging. While athletes with ID and without ID were perceived to be motivated by their sport progression, the athletic identity of the former is less clear. Further investigation is needed to better understand the athletic identity of athletes with ID and how they think and act within the sports context.

It seems that athletes without ID also participate in sports for physical and mental health reasons, as they want to get fit and relieve stress through sports, but this theme (‘Health-related reasons’) was not identifiable in athletes with ID. The difference could be partially due to the lack of understanding of their physical and mental conditions [39, 40]. Raising awareness of a tailored health management plan that would help people with ID to communicate their health-related issues better, could potentially encourage sustained engagement in an inclusive sport environment [39].

Our second aim was to gain a better understanding of the coaching motivations of coaches. This could inform us the extent to which they contribute to the motivations of athletes and the coaching practices of coaches. Our results have demonstrated that there are inherent reasons that aspire coaches of athletes with and without ID to coach their chosen sport (‘Personal and Professional Development’ and ‘Help others’ theme). Being driven by internal rewards can lead to greater satisfaction in and commitment to their coaching role [35, 41], which we have repeatedly seen from the interviews. These coaching motivations could act as a societal facilitator according to the SRM [33], as they empower the athletes, make them feel more competent, and encourage them to participate in sports and reach their goals [12].

This led us to dive more into the coaches’ practices (third aim) and to explore their relationships with the motivations of athletes and coaches. Specifically, all of our coaches’ intrinsic reasons for coaching a sport (‘Personal and Professional Development’ and ‘Help others’ themes) could explain why they emphasize the importance of connecting with their athletes (‘Build Meaningful Relationships’ theme), adapting their behaviour towards them (‘Behaviour adaptations’ theme) and developing their life skills (‘Psychological and Life skills development’ theme) [42, 43]. The coaching practices also seem to address the intrinsic motivations of athletes (‘Social Interaction’ and Positive Emotions’ themes). This could be crucial, as coaches who meet athletes’ needs and motivations, give them options and facilitate their development, can cultivate a fertile ground for their athletes’ long-term participation in sports [4]. Importantly, the similarities in motivations of athletes coaches’ motivations as well as the motivations and practices of coaches between the two groups (ID and non-ID) could facilitate sports inclusion of people with ID. For instance, athletes’ common motivations to progress in their sports, socialise with each other, and experience enjoyment through sports can promote group cohesion, psychological collectivism, and athletes’ engagement in sports [44]. The common motivations of coaches (e.g., help athletes reach their potential) and practices (e.g., adapt their practices to athletes’ needs and abilities) could mean that they require fewer fundamental adaptations in their attitudes towards different populations, which can facilitate a smoother transition from a segregated to an inclusive sports environment [4]. However, some barriers in the practices of coaches were identified.

A potential barrier to mainstreaming is the lack of emphasis on inclusivity from coaches of non-ID athletes while coaches of athletes with ID are trying to be supportive, develop athletes with ID life skills and engage them as much as they can in sports (the ‘Performance-focused vs ‘Participation-focused’ theme). The difference could stem from the fact that coaches of athletes without ID might be more motivated by ‘Career aspirations’ which might have led to a performance-focused approach, compared to the participation-focused approach that encourages inclusivity, adopted by the coaches of athletes with ID. This difference in coaching motivation and coaching behaviour might suggest that coaches of athletes with and without ID perceived themselves (or their coaching identity) differently, with coaches of athletes with ID to adopt mainly a mentor rather than a sport-coaching role [45]. While coaches of athletes without ID may overlook the importance of inclusivity in a mainstreaming environment, coaches of athletes with ID may underestimate the athletic identity that people with ID may wish to develop [46]. We can only speculate that a reason for the difference in the coaching approach is the ableist mentality that leads the coaches to adopt different sporting standards of success for athletes with and without ID [47]. To achieve a balance between performance-focused and participation-focused approaches, be it for athletes with or without ID, it is crucial for coaches to listen to their athletes’ sporting aspirations and respond to them accordingly. This could help coaches and athletes to set more appropriate and desirable goals and plan realistically how to achieve them [5]. With flexibility in coaching focus in place, mainstreaming initiatives are likely to be achievable and sustainable [1].

Another potential societal barrier for introducing mainstreaming in sport could come from overprotective parents. Their overprotectiveness may arise from the prejudice that they experience from other parents, as they are considered responsible for the disability of their child [48, 49]. All these factors confirm the social oppression that arises from the negative interactions between people with and without ID [33]. Lack of understanding the needs of athletes and unhelpful societal attitudes towards athletes with disabilities are quite common in sports, and act as barriers for the long-term participation of athletes with ID in an inclusive environment [19]. While the coaches in our interviews had recognized the importance of adapting their sessions and their attitudes in order to avoid conflicts with their athletes (e.g., the behavioural problems of the athletes with ID during competition that this study found, probably due to increased stress and anxiety of the competitive environment [15], education to parents and coaches of people without ID is also vital. By raising understanding of the needs and challenges faced by parents of people with ID, stigma against parents of people with ID can be prevented, and coaches can acquire knowledge in their approach towards athletes with ID in inclusive practices. For mainstreaming to be substantiated in sports and fair inclusive participation of people with ID in it, it is critical to address these issues through multisectoral campaigns that will promote disability sports participation awareness and offer inclusive coaching training opportunities [50]. Within and beyond research, co-producing initiatives to promote disability sports and inclusivity, with athletes with and without disability, as well as with their social agents, could generate rich and novel knowledge, deliver meaningful strategies that can positively influence the lives of people with and without, ID and further support public health campaigns [51].

### Limitations and future studies

The study presents some limitations that need to be mentioned. First, this study was based only on coaches’ experiences of athletes’ motivations and did not include athletes’ own perceptions. We chose to focus only on coaches due to the critical role of coaches in the sports engagement of athletes [21] and the challenges in interviewing people with ID [52]. However, the cognitive limitations of athletes with ID could lead their coaches to misinterpret their motivations and behaviours [11]. To gain a better understanding of the sport of athletes with ID, future work should directly explore the sports experiences of this population. Future research should also consider the combination of interviewing and observational methods [53]. This approach could elicit more diverse views and opinions related to sports for people with ID and the role of the social environment. Moreover, this study did not take into consideration the varying cognitive ability and age (adolescents and adults) of athletes with and without ID. Due to the potential behavioural and need differences that may exist within the population [54], future studies should investigate the motivational and coaching practices differences between athletes with mild and severe ID.

## Conclusions

In summary, this paper has highlighted the challenges in promoting inclusivity in the sports environment through understanding the motivations of athletes and coaches from the perspectives of coaches of athletes with and without ID. The intricate relationship between coaches’ motivations and their coaching practice, as well as the role of other social agents of the athletes were also explored. Based on the many similarities in the practices of coaches and the motivations of coaches and athletes with and without ID, we are cautiously optimistic that the numerous individual and societal facilitators can promote long-term sports participation of athletes with ID through enhanced awareness of inclusivity. However, more work is needed to overcome potential existing sports participation barriers, for example, ableism, addressing athletes’ sports-related needs and motivations, and educating athletes without ID and their social agents challenges faced by athletes with ID. The findings can inform stakeholders about the necessity of a multidisciplinary collaboration (e.g., governing and community bodies, coaches, families, researchers) that will further support athletes with ID and will offer them enhanced opportunities to participate (and maintain) in sports.
